# Comparison of door-to-door and fixed-point delivery of azithromycin distribution for child survival in Niger: A cluster-randomized trial

**DOI:** 10.1371/journal.pgph.0002559

**Published:** 2023-11-15

**Authors:** Ahmed M. Arzika, Ramatou Maliki, Abdou Amza, Alio Karamba, Nasser Gallo, Bawa Aichatou, Ismael Issa Sara, Diallo Beidi, Laminou Maliki Haroun, Farissatou Oumarou, Elodie Lebas, Brittany Peterson, Emily Colby, William Nguyen, Zijun Liu, Meagan C. Fitzpatrick, Benjamin F. Arnold, Thomas M. Lietman, Kieran S. O’Brien, the AVENIR Study Group

**Affiliations:** 1 Centre de Recherche et Interventions en Santé Publique, Birni N’Gaoure, Niger; 2 Programme Nationale de Santé Oculaire, Niamey, Niger; 3 Francis I. Proctor Foundation, University of California, San Francisco, California, United States of America; 4 Center for Vaccine Development and Global Health, University of Maryland School of Medicine, Baltimore, Maryland, United States of America; 5 Department of Ophthalmology, University of California, San Francisco, California, United States of America; 6 Department of Epidemiology and Biostatistics, University of California, San Francisco, California, United States of America; 7 Institute for Global Health Sciences, University of California, San Francisco, California, United States of America; Washington University School of Medicine, UNITED STATES

## Abstract

Recent evidence indicates mass azithromycin distribution reduces under-5 mortality. This intervention is being considered for child survival programs in high mortality sub-Saharan African settings. The delivery approach used in prior studies required a full-time census and distribution team, which is not feasible for most programs. To determine the optimal programmatic approach to delivery, this study aimed to compare treatment coverage, costs, and acceptability of different delivery approaches with existing community health workers (CHWs). This cluster-randomized trial included rural and peri-urban communities in Dosso, Niger (clinicaltrials.gov, NCT04774991). A random sample of 80 eligible communities was randomized 1:1 to biannual door-to-door or fixed-point delivery of oral azithromycin to children 1–59 months old over 1 year. Data analysts alone were masked given the nature of the intervention. The primary outcome was community-level treatment coverage defined as the number of children treated recorded by CHWs divided by the number of eligible children determined using a post-distribution census. Costs were monitored through routine administrative data collection and micro-costing. The census included survey questions on intervention acceptability among caregivers, community leaders, and CHWs. After randomization, 1 community was excluded due to inaccuracies in available administrative data, resulting in 39 communities receiving door-to-door delivery. At the second distribution, community-level mean treatment coverage was 105% (SD 44%) in the door-to-door arm and 92% (SD 20%) in the fixed-point arm (Mean difference 13%, 95% CI -2% to 28%, *P*-value *=* 0.08). The total cost per dose delivered was $1.91 in the door-to-door arm and $2.51 in the fixed-point arm. Indicators of acceptability were similar across stakeholder groups in both arms, with most respondents in each group indicating a preference for door-to-door. Overall, door-to-door delivery is the preferred approach to azithromycin distribution in this setting and might reach more children at a lower cost per dose delivered than fixed-point.

**Trial Registration**: clinicaltrials.gov NCT04774991.

## Introduction

Substantial heterogeneity in the global burden of under-5 mortality (U5MR) persists, with the West and Central Africa region reporting 95 deaths per 1000 livebirths in 2019, 19 times higher than the U5MR reported in high income settings [1, 2]. These settings are estimated to require unprecedented rates of decline to achieve the Sustainable Development Goal targets for under-5 mortality by 2030 [1–3]. Azithromycin distribution has been demonstrated to be an effective approach to reducing child mortality. The *Macrolides Oraux pour Réduire les Décès avec un Oeil sur la Résistance* trial (MORDOR) found that biannual distribution of oral azithromycin to children 1–59 months old reduced mortality by 14% in Malawi, Niger, and Tanzania after 2 years [4]. A longer-term follow-up of the Niger site and a pooled analysis of available data on azithromycin distribution and child mortality from several sub-Saharan African settings echoed these findings [5, 6]. In 2020, the World Health Organization (WHO) released conditional guidelines suggesting this intervention be considered in some high mortality settings in sub-Saharan Africa [7].

As this intervention transitions from trial to program settings, questions remain about optimal approaches to delivery. The mortality studies conducted to date used a more resource-intensive approach than would be feasible for programmatic delivery. The studies involved full-time, dedicated study teams to sensitize communities, administer the intervention, and track progress through biannual door-to-door visits throughout the study areas. Programs, however, might work within existing health delivery systems and involve teams that are managing multiple priorities. The objective of this study was to evaluate common programmatic approaches to intervention delivery using existing community health workers (CHWs). We used an implementation science framework with a cluster randomized trial design to compare community-based door-to-door and fixed-point delivery of azithromycin distribution for child survival across several indicators, including coverage, costs, acceptability, appropriateness, and fidelity at multiple stakeholder levels [8]. A cluster randomized design was used given the community-based nature of the intervention. We hypothesized that door-to-door delivery would have higher coverage, higher costs, and similar acceptability, appropriateness, and fidelity compared to fixed-point delivery.

## Materials and methods

### Trial design

This parallel arm cluster randomized trial randomized 80 rural communities in Niger to receive biannual azithromycin distribution by CHWs using door-to-door or fixed-point delivery. The unit of randomization was the *grappe*, an administrative unit defined in Niger and hereafter referred to as “community.” The study period included two distributions approximately 6 months apart, with outcomes measured at the second distribution. The present implementation study was conducted concurrently with a larger effectiveness trial in the same area [9]. The study design and reporting followed TIDieR and CONSORT guidelines (S1 and S2 Checklists) [10, 11].

### Ethics and oversight

Approval for this trial was obtained from the Institutional Review Boards at the Niger Ministry of Health (*Comité Nationale Éthique pour la Recherche en Santé*, N 041/2020/CNERS) and the University of California, San Francisco (19–28387). At the community level, verbal consent was obtained from community and local health center leaders before study activities commenced. At the individual level, informed consent was obtained from caregivers before study procedures were conducted, with written consent obtained for children 30–42 days old and verbal consent obtained for children older than 42 days. Written consent was required for the younger age group given the potential risk of macrolide-associated infantile hypertrophic pyloric stenosis that has been suggested in studies conducted in other settings [12]. An independent Data and Safety Monitoring Committee was empaneled to review the study protocol, procedures, and participant safety in the concurrent effectiveness and implementation trials. The present trial was registered at clinicaltrials.gov (NCT04774991). Additional information regarding the ethical, cultural, and scientific considerations specific to inclusivity in global research is included in the Supporting Information (S1 Questionnaire).

### Setting and participants

Eligibility criteria for communities included location in the Dosso region, population between 250 and 2500 inhabitants according to the 2012 national census, distance greater than 5 kilometers from the district headquarters town, and consent of community leaders. Communities indicated as urban on the national census were excluded. The same community-level criteria defined eligibility in the concurrent trial [9]. From this overall pool of eligible communities, simple random samples were used to select communities for participation in each trial, enabling generalizability of results across trials. At the individual level, children 1–59 months of age weighing at least 4 kg whose caregivers provided informed consent were eligible for inclusion. Recruitment for the overall project began in November 2020.

### Interventions

In both arms, the intervention consisted of biannual distribution of a single dose of oral azithromycin to children 1–59 months by existing *relais communautaires* (CHWs). CHWs are chosen by the communities they serve and they live in or near the communities themselves, facilitating a variety of health education and care activities, with a particular focus on maternal and child health. As *Centres de Santé Integrés* (CSIs, primary health care centers) oversee the work of the CHWs, the study team trained CSI leaders to conduct the interventions and these leaders in turned conducted the training of the CHWs themselves. All trainings consisted of one day of lecture and hands-on practice determining eligibility, obtaining consent, determining dose, administering the intervention, and completing the paper data collection form. The study team conducted supervision visits to monitor the conduct of the interventions.

In both arms, a sensitization campaign was performed by members of the study team, district health communication team, and the CSI leaders before distributions began. This included a community meeting at the home of the *chef du village* (community leader) to present the purpose and nature of the study and answer questions. Communities were notified of the distribution date one day before it occurred by the leader of the relevant CSI.

Two CHWs participated in the distributions. Azithromycin (Zithromax) was administered as oral suspension at 20 mg/kg of bodyweight up to the adult dose of 1 g. Dose was determined by weight for children 1–11 months of age or those unable to stand using an electronic hanging scale (ADE M111600, Hamburg, Germany). A table with the doses corresponding to possible weight ranges was provided to each CHW to facilitate the process. For children older than 11 months, dose was determined with the height-based dosing tape used by Niger’s trachoma program. The azithromycin powder for oral suspension was reconstituted using bottled water and each dose was measured into a new dosing cup or syringe for children too young to drink from a dosing cup. Each CHW recorded the number of doses administered by sex and age category (1–11 month or 12–59 months), reasons for ineligibility or refusal, and the number of days required for the distribution. CHWs were asked to complete each community’s distribution within 3 days for the first round and in 2 days for the second round, though they were able to work as many days as required to cover the community.

In communities randomized to receive the door-to-door delivery approach, CHWs visited each household in the community on foot and asked if children 1–59 months of age resided there to identify eligible children for inclusion. In communities randomized to fixed-point delivery, the CHWs worked with local leaders to identify an appropriate central location for the distribution. Common locations included outside the home of the community leader and the local mosque. A table and chairs were set up outdoors at the central location and the mobilizers would remind residents of the date, time, and location of the distribution in advance. Caregivers and children presented to the central location and the CHW then determined eligibility.

In both arms, all study team members and CHWs wore face masks during training and intervention activities and sanitized or washed hands with soap and water between each administration. All intervention activities took place outdoors with distancing of 6 feet maintained where possible.

A door-to-door census was conducted after each distribution to determine the total number of eligible children 1–59 months old. The census conducted after the second distribution included a survey of stakeholder perceptions of the accessibility, acceptability, and feasibility of the intervention. Stakeholders included caregivers, CHWs, and community leaders. The census was conducted within a 2-week window after the distributions so as not to influence the conduct of the intervention and to order include the post-treatment survey. Verbal informed consent was obtained at the household and individual level for participation in the survey. Eligible caregivers were those with a child eligible for the distribution and eligible CHWs were those who participated in the distribution. All community leaders were invited to participate. Eligibility for all survey groups included presence in the household at the time of the census.

### Adverse events

During the consent process, CHWs informed caregivers of the common adverse events experienced with azithromycin, including adnominal pain, nausea, vomiting, diarrhea, and skin rash, as well as the possibility of severe adverse events including infantile hypertrophic pyloric stenosis. Caregivers were instructed to report any adverse events experienced in the 28 days following treatment to the CHW, who then reported to the study team by phone. Serious adverse events were reported by email to Pfizer and to the medical monitors, which included one Niger-based pediatric infectious disease specialist and one US-based ophthalmologist with extensive experience in azithromycin distributions for trachoma.

### Data collection

Baseline characteristics of communities and CHWs involved routine data available from CSIs on total and target population size, distance to CSI and distance to the nearest town. Data were also collected on the CHWs themselves during training. CHWs recorded information about treatment administration on paper data collection forms. The forms included the number of doses delivered by age and sex, reasons for not treating, and adverse events. Forms were reviewed for accuracy and completeness during supervision and then entered into CommCare (CommCare by Dimagi) by the study team once distributions were completed. The supervision team also collected data on fidelity of the intervention rollout directly into a CommCare mobile application. The census was conducted by a separate study team using a CommCare mobile application that included data collection on household location, number and demographics of household members, and a survey of stakeholder perceptions of the accessibility, acceptability, and fidelity of the intervention implementation. Identifiable information was collected during the census and removed for analysis.

### Outcomes

All outcomes were defined at the community level using a combination of the Proctor Implementation Outcomes and RE-AIM frameworks and were assessed at the second distribution [8, 13, 14]. The primary outcome was community-level treatment coverage (reach), defined as the number of doses administered according to the data collection form divided by the total eligible population according to the post-distribution census. The separation of data collection for the numerator and denominator used to calculate treatment coverage resulted in uncertainty in the denominator since the census may have captured a slightly different population than the one present for the distribution. This resulted in coverage of > 100% for some communities. To evaluate the impact of this uncertainty, two alternative outcome definitions were used: one using the CSI estimate of the under-5 population as an alternative denominator and another capping treatment coverage estimates at 100%. Exploratory subgroup analyses included those to assess equity of treatment coverage by sex, age, community ethnicity, community population size, and community distance to the nearest health center.

Secondary outcomes included costs. As costs were anticipated to be higher for the first distribution given the additional expense associated with launching a new program, cost outcomes focused on the second distribution but are presented for both distributions. Costs were estimated through micro-costing, using known costs for personnel, training, and supplies required to distribute azithromycin at the community level. All costs were validated by comparing micro-costing estimates with routine administrative expenditure forms. Although for the trial each CHW was guaranteed a standard payment regardless of the number of days worked, for our micro-costing we included a per diem payment for CHW based on the actual number of days each CHW worked, which better reflects real-world compensation for CHW in Niger. As a sensitivity analysis, we also estimated cost outcomes using the standard payment. We calculated the cost per dose delivered by arm and round, with the main analyses presenting overall costs. Overall costs were chosen for main analyses as this method implicitly weights communities based on size and is most relevant to overall budget considerations. Community-level costs are presented as a sensitivity analysis, as these measures allow us to capture and quantify cost variability between communities.

Other secondary outcomes included an alternative indicator of coverage (reach), acceptability, appropriateness, and fidelity of the intervention at the participant and provider levels as measured through the post-distribution survey after the second distribution. The alternative indicator of coverage was the caregiver self-report of uptake of the intervention for eligible children. Acceptability of the intervention was defined as stakeholder perception that the conduct of the intervention was satisfactory and that the intervention was accessible. Appropriateness was defined as stakeholder perception of the utility of the intervention and approach to delivery. Fidelity of the intervention was defined as the degree to which the intervention was conducted as intended in the protocol and was measured by adherence to the protocol steps recorded during supervision visits and survey questions confirming the effectiveness of the publicity. Survey questions were drafted in French and discussed among the full study team to confirm they captured the intended outcome and census workers were trained to ask each question in local languages. A summary of how the survey questions map to the outcome definitions is included in S1 Appendix.

### Randomization and masking

Eighty communities were randomly selected from the eligible pool of communities in the Dosso region. The randomization sequence was prepared by the UCSF biostatistician in R (R Foundation for Statistical Computing, Vienna, Austria). Communities were randomized equally to the two arms in a 1:1 ratio without stratification or matching. Community selection and allocation were shared with the Niger study team who enrolled communities and ensured communities received their assigned intervention. Given the nature of the intervention, participants, investigators, and study personnel implementing the intervention were not masked to allocation. Outcome assessors conducting the post-distribution census were not informed of the intervention but were not able to be completely masked since responses to the survey questions could reveal allocation. The data analyst conducting data management and analyses was masked to allocation.

### Statistical methods

Assuming a community-level standard deviation of 8.2% based on prior treatment coverage data from the MORDOR trial [4], including 40 communities per arm (80 communities total) was expected to provide 80% power to detect an absolute difference in mean community treatment coverage of 5% at an alpha of 0.05. Baseline characteristics were summarized descriptively and compared with t-tests or Wilcoxon rank sum tests for continuous variables and Fisher exact tests for categorical variables. The primary analysis aimed to compare the cluster-level mean treatment coverage by arm, using permutation to estimate the *P-*value. The sensitivity analyses using alternative definitions were conducted in the same manner, using local health center estimates of eligible population or treatment coverage capped at 100%. An additional sensitivity analysis used the Kolmogorov-Smirnov test to compare the eligible population distributions in the two arms. As the *P*-value from this test was > 0.05, we assumed the samples were drawn from the same distribution and proceeded with a comparison of the cluster-level mean number of children treated by arm using permutation as defined for the primary analysis. This allowed for a comparison of children treated without the uncertainty inherent in the denominator. Sensitivity analyses were conducted comparing the community-level median for each outcome using permutation as well. Treatment coverage for all subgroups were summarized descriptively and community-level subgroups were analyzed using linear regression models with log-transformed number of children treated as the outcome, indicators for treatment arm and subgroup, log-transformed number of eligible children as a covariate, and an interaction term between arm and subgroup. Overall costs were summarized as total costs per arm divided by total doses delivered by arm. Analyses of survey outcomes and alternative cost outcomes were conducted similarly to the coverage analyses. Analyses were conducted in R version 4.2.2.

## Results

**Fig 1:**
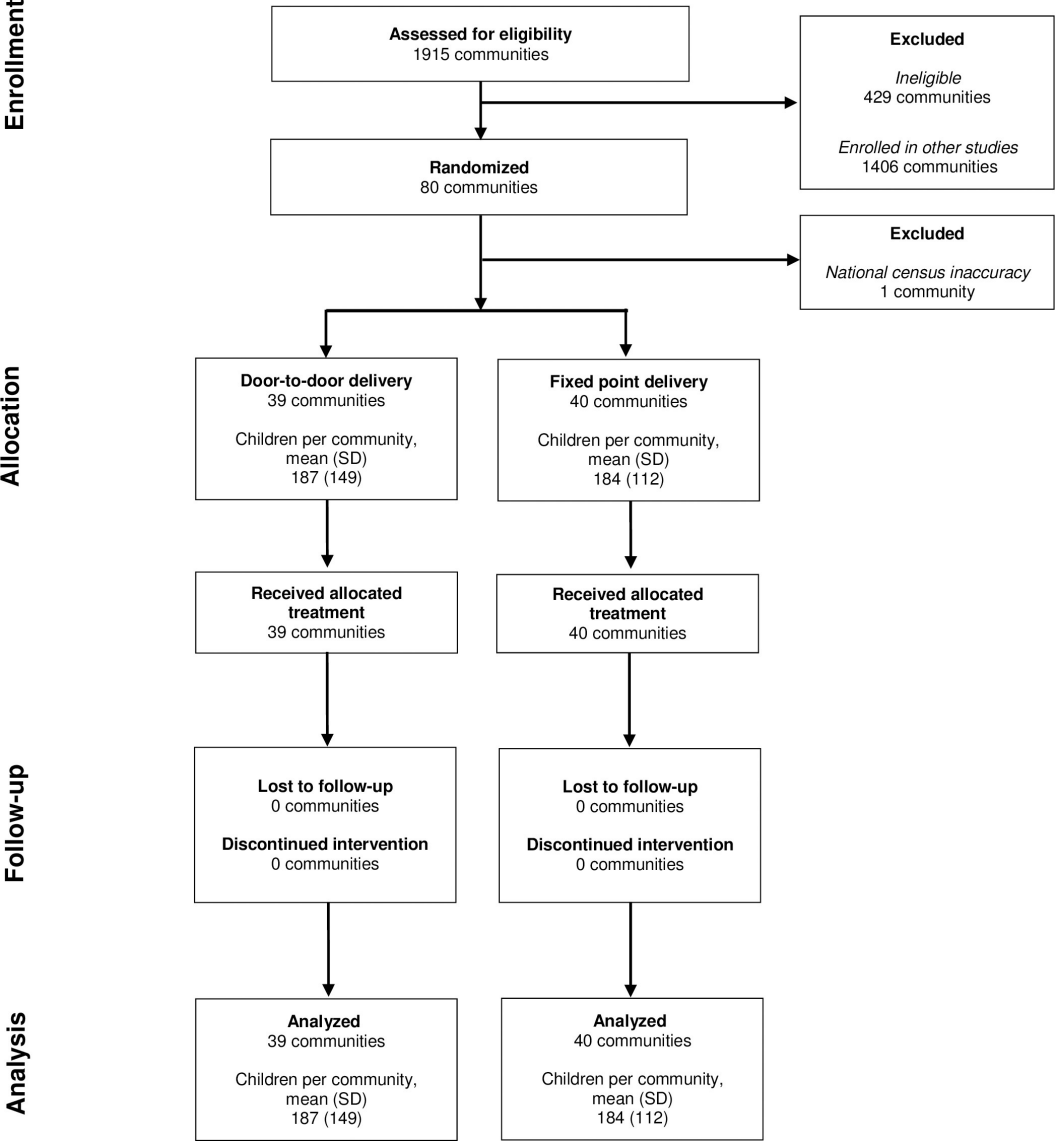
Participant flow diagram (Consolidated Standards of Reporting Trials, CONSORT).

In November 2020, 1,915 communities in the Dosso region were assessed for eligibility for the main trial (Fig 1). Of these, 429 did not meet eligibility, 1,406 were randomly assigned to and enrolled in other studies, and 80 were included in the present trial and randomized to receive door-to-door or fixed-point delivery of azithromycin. After randomization, 1 community was excluded as the community was identified as having been listed twice in the national census data and already treated as part of other studies. The remaining 79 communities received their allocated delivery modality and were included in analyses. According to administrative estimates from CSIs, the mean number of children per community was 187 (standard deviation [SD] 149) in the door-to-door arm and 184 (SD 112) in the fixed-point arm. The estimated eligible population varied by data source (study census or administrative estimates) but was similar overall by arm (S1 Appendix).

Table 1 summarizes characteristics of included communities and their CHWs before distributions began. Overall, the mean distance to the nearest CSI was 8.4 km (SD 9.0). Most CHWs were male (79.3%) with an average age of 36.9 years (SD 11.4). CHWs had an average of 3.9 years of experience in the role (SD 4.4). No statistically significant differences in baseline characteristics were demonstrated between arms.

**Table 1:** Baseline characteristics of enrolled communities and community health workers.

Characteristic	Door-to-door	Fixed-point	Overall
	(N = 39)	(N = 40)	(N = 79)
Total population, n^1^	29,230	25,982	55,212
Mean overall population size (SD)^1^	750 (523)	650 (304)	699 (427)
Mean target population size (SD)^1^^,^^2^	187 (149)	182 (112)	186 (132)
Mean distance to nearest town, km (SD)^1^	28.9 (13.1)	27.7 (11.5)	28.3 (12.2)
Mean distance to primary health center, km (SD)^1^	8.4 (10.8)	8.3 (6.9)	8.4 (9.0)
Primary community ethnicity, n (%)			
Zarma	18 (45.0%)	18 (45.0%)	36 (45%)
Hausa	21 (52.5%)	18 (45.0%)	39 (48.8%)
Peul	0 (0.0%)	1 (2.5%)	1 (1.3%)
Other	1 (2.5%)	1 (2.5%)	2 (2.5%)
Multiple	0 (0.0%)	2 (5.0%)	2 (2.5%)
Mean age of community health workers (SD)^3^	37.5 (11.4)	36.3 (11.5)	36.9 (11.4)
Community health workers with female sex, n (%)^3^	9 (19.1%)	10 (22.2%)	19 (20.7%)
Community health workers education, n (%)^3^			
Primary	31 (66.0%)	28 (62.2%)	59 (64.1%)
Secondary or higher	11 (23.4%)	11 (24.4%)	22 (23.9%)
Other^4^	4 (8.5%)	6 (13.3%)	10 (10.9%)
Missing	1 (2.1%)	0 (0%)	1 (1.1%)
Mean years of experience as community health worker (SD)^3^	4.0 (4.0)	3.8 (4.8)	3.9 (4.4)

SD, standard deviation

^1^Population and distance estimates from primary health centers.

^2^Target population refers to children 1–59 months old and was estimated based on the number of children 3–59 months of age targeted for seasonal malaria chemoprevention and adding 3% to account for children 1–2 months of age.

^3^Overall, 92 community health workers participated in the study, including 47 in door-to-door communities and 45 in fixed-point communities. These are used as the denominators for the community health worker percentages presented for female sex and education.

^4^Other education level includes Korannic school.

The median number of days between the second distribution and the post-distribution census was 5 (inter-quartile range 3 to 7 days) overall and was similar by arm. Community-level mean treatment coverage at the second distribution was 105% (SD 44%) in the door-to-door arm and 92% (SD 20%) in the fixed-point arm (Table 2; Mean difference 13%, 95% CI -2% to 28%, permutation *P*-value *=* 0.08). Median treatment coverage was 94% in each arm (Table 2; 95% CI 83–103% in door-to-door and 78–104% in fixed point, *P-*value 0.38). Sensitivity analyses using other alternative outcome definitions were also unable to detect a difference in coverage by arm (Table 2). The distribution of treatment coverage was similar in both arms, with the exception that the door-to-door approach resulted in more communities reporting a greater number of children treated than censused compared to fixed-point, resulting in treatment coverages of more than 100% (S1 Appendix). Coverage was similar across all subgroups (S1 Appendix).

**Table 2:** Community-level treatment coverage by arm in Round 2.

Analysis	Door-to-door	Fixed-point	Difference	*P*-value
	N = 39	N = 40	(95% CI)	
***Primary analysis***				
Mean coverage (SD)^1^	105% (43%)	92% (20%)	13% (-2% to 28%)	0.08
***Sensitivity analyses***				
Alternative mean coverage (SD)^2^	105% (84%)	85% (84%)	20% (-19% to 58%)	0.31
Capped mean coverage (SD)^3^	90% (11%)	87% (15%)	3% (-3% to 9%)	0.30
Mean number treated (SD)^4^	158 (117)	120 (60)	38 (-3 to 79)	0.07
Median coverage (IQR)^2^	94% (83% to 103%)	94% (78% to 104%)	0% (-12% to 14%)	0.38
Alternative median coverage (IQR)^5^	82% (60% to 114%)	72% (40% to 100%)	10% (-10% to 34%)	0.33
Median number treated (IQR)^4^	111 (82 to 192)	105 (71 to 154)	6 (-26 to 51)	0.35

^1^Pre-specified primary outcome defined as coverage calculated by dividing the number of children treated as recorded during delivery by the estimated number of eligible children as recorded on the post-distribution census.

^2^Sensitivity analysis defined as coverage calculated by dividing the number of children treated as recorded during delivery by the estimated number of eligible children according to primary health centers.

^3^Sensitivity analysis capping treatment coverage estimates at 100%.

^4^Sensitivity analysis comparing the mean number of children treated as recorded during delivery after Kolmogorov-Smirnov test comparing census and health center estimates of eligible children by arm resulted in *P-*values of 0.9 and 0.8, respectively.

^5^Sensitivity analysis defined as coverage calculated by dividing the number of children treated as recorded during delivery by the estimated number of eligible children according to primary health centers.

Two serious adverse events in the fixed-point arm were reported to the study team during the second distribution. Both children experienced diarrhea and vomiting 24–48 hours after azithromycin administration. Both were admitted to the CSI with dehydration and treated with metoclopramide, oral rehydration solution, and zinc and were released the same day as symptoms subsided. In the first round of distribution, one fixed point grappe did not receive distributions as part of this study due to outdated census information but the eligible population did receive distributions in the second round as anticipated.

Estimates of the overall costs associated with each delivery modality are shown in Table 3. In both arms, the first distribution incurred greater costs associated with both training and delivery than the second distribution. At the second distribution, 6,144 doses were delivered in the door-to-door arm and 4,781 doses were delivered in the fixed-point arm, resulting in a delivery cost per dose of $0.68 in the door-to-door arm and $0.91 in the fixed-point arm. When including training costs, the total cost per dose delivered at the second distribution was $1.91 in the door-to-door arm and $2.51 in the fixed-point arm. Community-level costs are presented in S1 Appendix, as well as a sensitivity analysis evaluating costs under the scenario where CHWs are provided a standard payment regardless of days actually worked.

**Table 3:** Estimates of overall costs and costs per dose delivered by arm.

	Round 1		Round 2	
	Door-to-door	Fixed-point	Door-to-door	Fixed-point
**Number of days worked by CHWs (mean, SD)** ^**1**^	1.7 (0.8)	1.8 (0.8)	1.5 (0.6)	1.5 (0.6)
**Costs**				
Training	$11,828.18	$11,828.18	$7,566.36	$7,610.00
Delivery	$5,439.98	$5,431.05	$4,161.58	$4,367.56
Total	$17,268.16	$17,259.24	$11,727.94	$11,977.56
**Outcome**				
Doses delivered	5,800	5,326	6,144	4,781
**Cost/outcome**				
Delivery cost/dose delivered	$0.94	$1.02	$0.68	$0.91
Total cost/dose delivered	$2.98	$3.24	$1.91	$2.51

SD, standard deviation

^1^The study team pre-specified a fixed number of distribution days for the CHWs to target but CHWs could work as many days as required to complete the distribution. Shown here are the actual days worked by CHWs.

**Fig 2:**
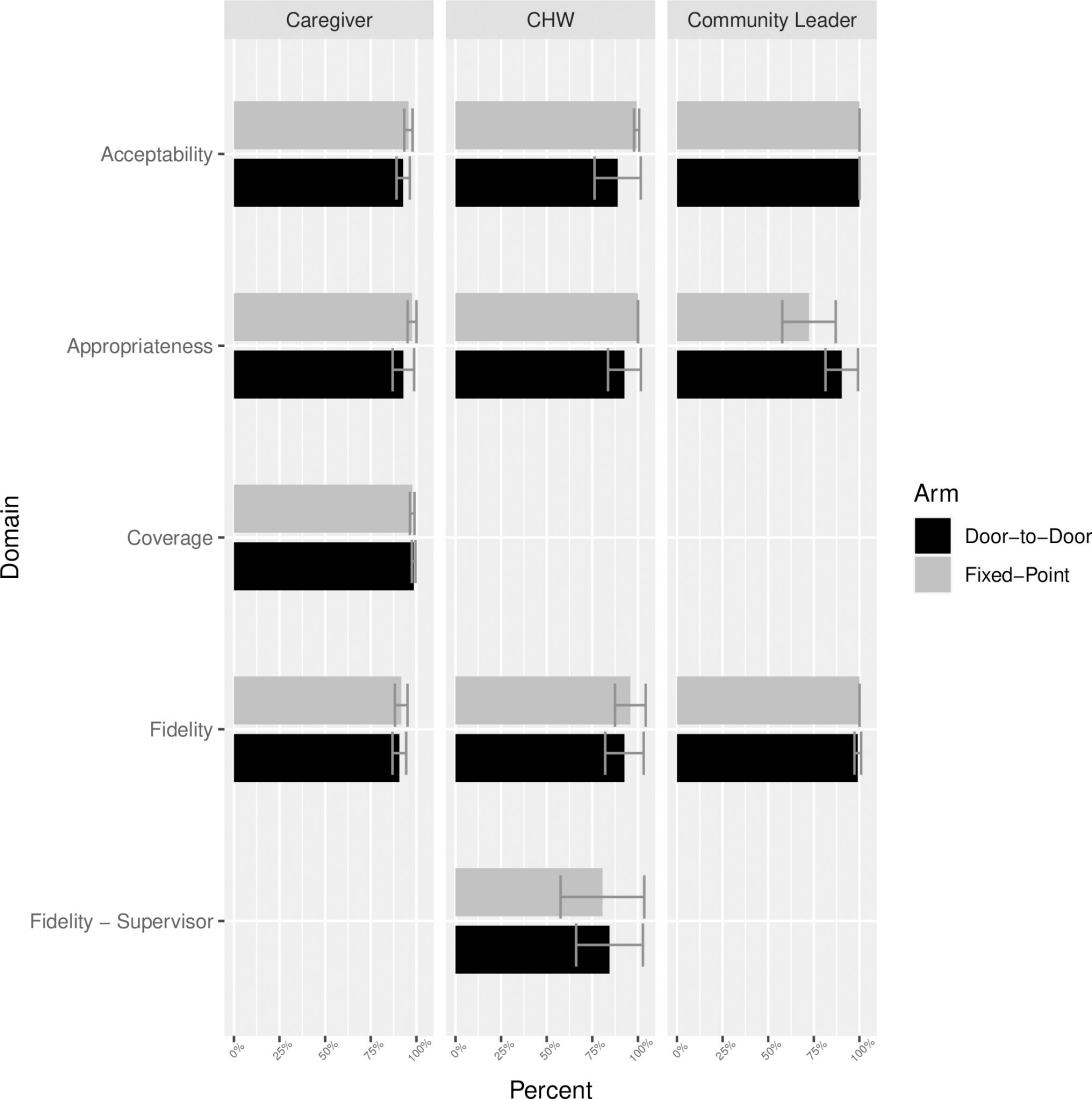
Community-level summary of implementation outcomes assessed by stakeholder surveys. Surveys were conducted among caregivers of eligible children, community health workers (CHWs), and community leaders in both arms to evaluate coverage (reach) acceptability, appropriateness, and fidelity of the intervention after delivery of the Round 2 distribution. The alternative indicator of coverage was assessed only among caregivers. Fidelity was assessed by survey for all groups and also by supervisor visits for the CHWs.

Fig 2 displays results of the stakeholder surveys for each domain of interest, with detailed survey responses reported in S1 Appendix. More than 99% (9954/9967) of eligible households in both arms agreed to participate and 99% (7080/7133), 93% (86/92), and 97% (97/99) of eligible caregivers, CHWs, and community leaders participated, respectively. Overall, indicators of fidelity, acceptability, and appropriateness were high and similar in each arm among each stakeholder group (Fig 2). Caregivers and community leaders indicated both modalities had a high degree of acceptability and appropriateness. When asked directly which approach they preferred, the majority of caregivers and community leaders in both arms preferred the door-to-door approach (90–93% in the door-to-door arm and 72–76% in the fixed-point arm preferred door-to-door; S1 Appendix), with reasons including that the approach ensures more children are treated and requires less personal travel for the caregivers. While the majority of CHWs in the door-to-door arm also preferred the door-to-door approach for the sake of the intervention’s goals, 49% of those in the fixed-point arm preferred fixed-point, in part because this modality simplifies the administration for the distributors (S1 Appendix). The majority of caregivers and community leaders indicated knowledge of the timing and purpose of the distribution, suggesting fidelity of the publicity effort to the protocol (S1 Appendix). Supervision visits indicated that most CHWs were administering azithromycin according to the protocol in both arms, with mean cluster-level adherence of more than 80% for each component (S1 Appendix). Although most caregivers in both arms reported participating in the distribution if they had an eligible child, a greater proportion of caregivers who did not participate said they were unaware of the intervention in the fixed-point arm compared to the door-to-door arm (10.0% vs 1.1%; S1 Appendix).

## Discussion

This cluster-randomized trial aimed to compare the implementation of door-to-door and fixed-point delivery of azithromycin distribution to increase child survival across a number of indicators, including treatment coverage (reach), costs, acceptability, appropriateness, and fidelity. Overall, treatment coverage was high for both approaches, with average cluster-level coverage reaching more than 80% in both arms and distribution rounds. Other operational studies of community mass drug administration delivery have found similarly high levels of coverage on average, though the range of coverage achieved can be quite wide [15–17]. Although an underlying goal of this study was to more closely mimic programmatic implementation, the presence of the study team for training and supervision may have contributed to the high coverage across most communities in this setting. We were unable to detect a difference in treatment coverage by arm given the degree of heterogeneity. Results suggested the possibility that door-to-door may result in a greater number of children treated, and more door-to-door communities reported treatment coverage > 100% than fixed-point communities, perhaps because this approach is able to include harder-to-reach households than fixed-point or even the census alone when not coupled with treatment. As anticipated, indicators of acceptability, appropriateness and fidelity of implementation were similar in both arms. Most caregivers and community leaders indicated a preference for the door-to-door approach, stating that this delivery modality is better able to reach all children and reduces the burden on caregivers.

The estimated overall costs across rounds were similar for the door-to-door and fixed-point delivery modalities given the similar overhead costs associated with each program and the pre-determined goal for distribution days per community. More doses were delivered in the door-to-door arm, however, resulting in lower costs per dose delivered. Studies of seasonal malaria chemoprevention have also suggested lower costs per dose delivered with the door-to-door approach [16, 18]. A systematic review of costs for mass drug administration in neglected tropical disease programs estimated costs to be less than $0.50 per dose delivered in 2015 for programs treating 100,000 people or more, but over $2.00 per dose for programs treating fewer than 10,000 people [19]. These costs primarily reflect programs that rely on local or school-based volunteers, which was not the case for our program which paid existing CHWs. Unit costs are also expected to decrease as the number of years of implementation increase [19]. Therefore, we anticipate that unit costs would decrease if all communities within eligible CSIs were treated over more rounds of distribution, and indeed we did see costs decrease between rounds in both arms, suggesting some improved efficiency with experience. Costs anticipated in a national scale-up of the program will be explored in future work. In addition, this program treated children 1–59 months of age but future programs may target children 1–11 months of age instead as recommended in the WHO guidelines [7]. The cost implications of this change in program target are currently being studied.

The strengths of this study include the cluster-randomized design, use of an implementation science framework to guide the development of outcome measurement, and the experienced study team and CHWs. Limitations include the challenge of population estimation and the gap between the distribution and census which resulted in inconsistencies between estimated number of children treated compared to the estimated number of children eligible. This resulted in estimates of treatment coverage of greater than 100%, which is common for similar programs.[15, 20] Typically, such programs use administrative estimates of coverage based on existing population estimates which can be unreliable [15, 20–24]. Despite conducting a separate population-based census to estimate the current eligible population, we still found inconsistencies in population estimates. Some mass drug administration programs using administrative coverage will also independently validate coverage estimates through post-distribution surveys of self-reported uptake [24, 25]. Our post-distribution survey indicated high coverage of > 90% in both arms, consistent with the primary analysis. In addition, it is also possible that neighboring communities learned of the distribution and brought their children for treatment despite not residing in the community, that children outside of the eligible age range were treated, and that residents were not at home during the census and so not counted in the denominator. Study team members and CHWs were trained to confirm residence in an attempt to reduce this possibility, but this process may not have completely eliminated the phenomenon. We attempted to address this challenge with sensitivity analyses, which produced similar results to the primary analysis. However, we found a greater variance in treatment coverage in this programmatic approach to delivery than anticipated based on prior studies and so the trial was underpowered to detect small effects, especially among subgroups. In addition, several domains of interest were estimated using subjective indicators collected through surveys. Although these were developed in close collaboration with Nigerien study team members to ensure cultural appropriateness and comprehensibility, these responses are subject to social desirability bias and may not fully reflect the spectrum of preferences. We anticipate these results are generalizable to similar settings in Niger targeted by mass drug administration programs and perhaps similar West African countries.

## Conclusions

Overall, we found similar treatment coverage, acceptability, appropriateness and fidelity of implementation of the door-to-door and fixed-point approaches to azithromycin distribution for child survival. Our results suggested door-to-door might enable greater numbers of children to be treated and lower the cost per dose delivered. Given this result along with the stakeholder preference for the door-to-door modality, door-to-door delivery may be the optimal approach to azithromycin distribution in this setting.

## Supporting information

S1 Checklist Consolidated Standards of Reporting Trials (CONSORT) checklist extension for cluster-randomized trials. (DOCX)

S2 Checklist Template for Intervention Description and Replication (TIDieR) checklist. (DOCX)

S1 Appendix Supplementary appendix. (DOCX)

S1 Questionnaire PLOS inclusivity in global research questionnaire. (DOCX)

S1 File Original IRB approval letters from UCSF and the Niger ethics committee in French and English. (PDF)
