# Supplementary material for: Comparison of door-to-door and fixed-point delivery of azithromycin distribution for child survival in Niger: A cluster-randomized trial
Source: PLOS Glob Public Health. 2023 Nov 15;3(11):e0002559. doi: 10.1371/journal.pgph.0002559 (PMC10651009; doi:10.1371/journal.pgph.0002559)

**S1 Appendix.** Supplementary Appendix for: Comparison of door-to-door and fixed-point delivery of azithromycin distribution for child survival in Niger: a cluster-randomized trial.

**Table of Contents.**

[Appendix Table 1. Map of survey questions to implementation science domains of interest. 2](#_Toc148262398)

[Appendix Table 2. Summary of overall, community-level mean, and community level median numbers of children treated and eligible by round and arm. 4](#_Toc148262399)

[Appendix Table 3. Community-level summaries of costs by arm and distribution round using actual number of days CHWs spent on distribution. 5](#_Toc148262400)

[Appendix Table 4. Community-level summaries of costs by arm and distribution round assuming a fixed number of days for distribution. 6](#_Toc148262401)

[Appendix Table 5. Full responses to stakeholder survey by domain for Caregivers. Responses shown as mean cluster-level summaries. 7](#_Toc148262402)

[Appendix Table 6. Full responses to stakeholder survey by domain for Community Health Workers. Responses shown as mean cluster-level summaries. 10](#_Toc148262403)

[Appendix Table 7. Full responses to stakeholder survey by domain for Community Leaders. Responses shown as mean cluster-level summaries. 13](#_Toc148262404)

[Appendix Fig 1. Histograms of the distribution of Round 2 treatment coverage by arm. 15](#_Toc148262405)

[Appendix Fig 2. Boxplots of treatment coverage by community-level subgroups. 16](#_Toc148262406)

[Appendix Fig 3. Boxplots of treatment coverage shown by individual level subgroups. 17](#_Toc148262407)

Appendix Table 1. Map of survey questions to implementation science domains of interest.^1^

| **Stakeholder Group** | **Domain** | **Survey questions** |
| --- | --- | --- |
| Caregiver | Acceptability | Was the program accessible? |
|  |  | Was the amount of time involved in participation reasonable? |
|  |  | Were the community health workers helpful? |
|  | Coverage (Reach) | Did you participate in the program by agreeing to have your child treated? If not, why not? |
|  | Appropriateness | Do you think this is a useful intervention for your community? |
|  |  | Which approach do you prefer, door-to-door delivery or fixed-point delivery? Why? |
|  |  | Are there other ways the program could be delivered that you would prefer?^2^ |
|  | Fidelity | Did a team recently visit your community to distribute azithromycin? |
|  |  | Do you know why azithromycin was distributed in your community? If so, why? |
|  |  |  |
| Community Leader | Acceptability | Was the program accessible? |
|  |  | Was the amount of time involved in participation reasonable? |
|  |  | Were the community health workers helpful? |
|  | Appropriateness | Which approach do you prefer, door-to-door delivery or fixed-point delivery? Why? |
|  |  | Are there other ways the program could be delivered that you would prefer?^2^ |
|  | Fidelity | Was a special meeting held to ask for your consent for your community’s participation? |
| Community Health Worker | Acceptability | Were the communities you worked in supportive of the program? |
|  | Appropriateness | Do you think this is a useful intervention for your community? |
|  |  | Which delivery approach do you think is better for communities, door-to-door delivery or fixed-point delivery? Why? |
|  |  | Which approach do you prefer as a community health worker, door-to-door delivery or fixed-point delivery? Why? |
|  |  | Are there other ways the program could be delivered that you would prefer?^2^ |
|  | Fidelity | Do you know why azithromycin was being distributed in your community? If so, why? |
|  |  | Did the supervision provide enough support for your effort? |
|  |  | Did the training provide you enough information to implement the protocol? |
|  | Fidelity - Supervisor | Was the intervention implemented according to the protocol?^3^ |

^1^Questions are presented here in a different order than the surveys themselves in order to group by domain and stakeholder group. Survey questions presented here in English. These questions were translated into French by bilingual French and English speakers in the US and Niger to ensure comprehension and appropriateness for cultural context. Study team members were trained on the French version of the questionnaires, and then trained to ask each question in local languages for the conduct of the survey itself.

^2^Question not included in final domain assessment given the nature of the responses.

^3^This question was assessed by a supervisor visit to review key steps of protocol implementation, including obtaining consent of the caregiver, determining dose by height, determining dose by weight, preparing the treatment, administering the treatment with a dosing cup, administering the treatment with a syringe, and completing the data collection form correctly. A community health worker was required to complete each of these steps correctly in order to receive a “Yes” response to this question.

# Appendix Table 2. Summary of overall, community-level mean, and community level median numbers of children treated and eligible by round and arm.

**2a.** Numbers treated by round and arm.^1^

|  | **Round 1** | | **Round 2** | |
| --- | --- | --- | --- | --- |
|  | **Door-to-door** | **Fixed-point** | **Door-to-door** | **Fixed-point** |
| Overall | 5,800 | 5,326 | 6,144 | 4,781 |
| Community-level mean (SD) | 149 (98) | 137 (57) | 158 (117) | 120 (60) |
| Community-level median (IQR) | 121  (82, 194) | 122  (100, 170) | 111  (82, 193) | 105  (71, 154) |

**2b.** Estimates of eligible population according to study census^2^ and CSI estimates^3^ by arm.

|  | **Door-to-Door** | | | **Fixed-point** | | |
| --- | --- | --- | --- | --- | --- | --- |
|  | Census Round 1 | Census Round 2 | CSI target | Census Round 1 | Census Round 2 | CSI target |
| Overall | 7,788 | 6,028 | 7,294 | 6,530 | 5,505 | 7,544 |
| Community-level mean (SD) | 195 (145) | 155 (110) | 187 (149) | 163 (84) | 138 (77) | 184 (112) |
| Community-level median (IQR) | 144  (99, 209) | 107  (87, 200) | 130 (78, 254) | 146  (106, 210) | 123  (81, 182) | 149 (100, 244) |

CSI, Centre de Santé Integré; IQR, interquartile range; SD, standard deviation

^1^Numbers of children treated recorded by community health workers on paper data collection forms during distributions.

^2^Population-based census data collection conducted after each round of distribution.

^3^Estimates of target population provided by CSIs.

# Appendix Table 3. Community-level summaries of costs by arm and distribution round using actual number of days CHWs spent on distribution.

|  | **Round 1**  **Median (IQR)** | | **Round 2**  **Median (IQR)** | |
| --- | --- | --- | --- | --- |
|  | **Door-to-door** | **Fixed-point** | **Door-to-door** | **Fixed-point** |
| **Number of days worked by CHWs** | 1.7 (0.8) | 1.8 (0.8) | 1.5 (0.6) | 1.5 (0.6) |
| **Costs** |  |  |  |  |
| Delivery alone | $137.49  ($118.65, $152.58) | $141.27  ($115.43, $154.82) | $102.27  ($91.54, $111.48) | $ 99.87  ($92.31, $123.63) |
| Total (delivery + training) | $440.77  ($421.94, $455.86) | $444.56  ($418.72, $458.10) | $296.28  ($285.55, $305.49)) | $290.12  ($282.56, $313.88)) |
| **Cost/outcome** |  |  |  |  |
| Delivery cost/dose delivered^1^ | $1.15  ($0.74, $1.49) | $1.07  ($0.83, $1.3) | $0.88  ($0.55, $1.18) | $1.00  ($0.81, $1.33) |
| Total cost/dose delivered^2^ | $3.65  ($2.3, $5.17) | $3.65  ($2.65, $4.28) | $2.56  ($1.59, $3.6) | $2.84  ($2.02, $3.98) |

IQR, interquartile range

^1^*P-*value for comparison of community-level delivery cost per dose delivered by arm in Round 2 was 0.24.

^2^*P-*value for comparison of community-level total cost per dose delivered by arm in Round 2 was 0.53.

# Appendix Table 4. Community-level summaries of costs by arm and distribution round assuming a fixed number of days for distribution.

|  | **Round 1**  **Median (IQR)** | | **Round 2**  **Median (IQR)** | |
| --- | --- | --- | --- | --- |
|  | **Door-to-door** | **Fixed-point** | **Door-to-door** | **Fixed-point** |
| **Number of days CHWs were asked to target** | 3 | 3 | 2 | 2 |
| **Costs** |  |  |  |  |
| Delivery alone | $158.73  ($154.8, $170.18) | $159.84  ($151.79, $170.61) | $110.94  ($107.19, $120.47) | $116.94  ($110.49, $123.74) |
| Total (delivery + training) | $462.01  ($458.08, $473.47) | $463.13  ($455.08, $473.90) | $304.95  ($301.2, $314.47) | $307.19  ($300.74, $313.99) |
| **Cost/outcome** |  |  |  |  |
| Delivery cost/dose delivered^1^ | $1.36  ($0.86, $1.90) | $1.32  ($0.98, $1.55) | $0.98  ($0.65, $1.33) | $1.12  ($0.81, $1.55) |
| Total cost/dose delivered^2^ | $3.81  ($2.43, $5.62) | $3.80  ($2.8, $4.58) | $2.72  ($1.65, $3.71) | $2.93  ($2.05, $4.24) |

IQR, interquartile range

^1^*P-*value for comparison of community-level delivery cost per dose delivered by arm in Round 2 was 0.31.

^2^*P-*value for comparison of community-level total cost per dose delivered by arm in Round 2 was 0.54.

# Appendix Table 5. Full responses to stakeholder survey by domain for Caregivers. Responses shown as mean cluster-level summaries.

| **Domain** | **Survey Question** | **Door-to-Door**  **% (95% CI)** | **Fixed-point**  **% (95% CI)** | **Overall**  **% (95% CI)** |
| --- | --- | --- | --- | --- |
| Acceptability | Was the program accessible? | 95.7%  (92.8%, 98.6%) | 97.9%  (96.9%, 98.9%) | 96.8%  (95.3%, 98.3%) |
|  | Was the amount of time involved in participation reasonable? | 95.3%  (92.8%, 97.8%) | 96.4%  (94.3%, 98.5%) | 95.9%  (94.2%, 97.4%) |
|  | Were the community health workers helpful? | 98.1%  (97.1%, 99.1%) | 97.5%  (96.1%, 98.8%) | 97.8%  (97.0%, 98.6%) |
| Coverage (Reach) | Did you participate in the program by agreeing to have your child treated? | 98.5%  (97.5%, 99.5%) | 97.7%  (96.4%, 99.0%) | 98.1%  (97.3%, 98.9%) |
|  | *For those who responded they did not participate in the program:* Why not?^1^ |  |  |  |
|  | Child ineligible | 5.0%  (2.3%, 7.8%) | 10.5%  (2.3%, 18.8%) | 8.0%  (3.6%, 12.4%) |
|  | Intervention is not useful | 3.1%  (0%, 6.7%) | 3.3%  (0%, 7.5%) | 3.2%  (0.6%, 5.9%) |
|  | Concern about side effects | 1.5%  (0%, 3.3%) | 0%  (0%, 0%) | 0.7%  (0%, 1.6%) |
|  | Mother or child not present | 82.3%  (75.1%, 89.5%) | 76.1%  (65.9%, 86.3%) | 79.0%  (72.8%, 85.1%) |
|  | Did not know about intervention | 1.1%  (0%, 2.4%) | 10.0%  (2.9%, 17.1%) | 5.9%  (2.1%, 9.7%) |
|  | Was not visited to receive intervention | 7.0%  (1.5%, 12.5%) | 0%  (0%, 0%) | 3.3%  (0.6%, 5.9%) |
| Appropriateness | Do you think this is a useful intervention for your community? | 92.9%  (87.0%, 98.8%) | 97.6%  (95.2%, 100%) | 95.3%  (92.1%, 98.4%) |
|  | Which approach do you prefer, door-to-door delivery or fixed-point delivery? *Percentage of participants indicating preference for door-to-door shown.* | 92.8%  (89.8%, 95.8%) | 76.1%  (67.8%, 84.4%) | 84.4%  (79.6%, 89.1%) |
|  | *For those who responded with preference for door-to-door delivery:* Why do you prefer that approach?^3^ |  |  |  |
|  | The method makes administering azithromycin easier | 25.2%  (19.3%, 31.2%) | 27.0%  (21.7%, 32.4%) | 26.2%  (22.3%, 30.0%) |
|  | Less personal travel required to receive treatment | 23.4%  (18.2%, 28.6%) | 22.2%  (17.8%, 26.6%) | 22.8%  (19.4%, 26.1%) |
|  | The method is more able to ensure all children are treated | 33.5%  (26.8%, 40.1%) | 32.7%  (26.2%, 39.3%) | 33.1%  (28.5%, 37.6%) |
|  | The method avoids possible conflict | 17.8%  (14.7%, 20.9%) | 18.1%  (13.9%, 22.3%) | 18.0%  (15.4%, 20.5%) |
|  | The chef du village influenced my decision | 0.1%  (0%, 2.1%) | 0%  (0%, 0%) | 0.04%  (0%, 0.1%) |
|  | Don’t know | 0.09%  (0%, 0.2%) | 0.04%  (0%, 0.03%) | 0.1%  (0%, 0.1%) |
|  | Other | 0.02%  (0%, 0.02%) | 0%  (0%, 0%) | 0.03%  (0%, 0.09%) |
|  | Are there other ways the program could be delivered that you would prefer? *Percentage of participants responding “Yes” is shown.* | 0%  (0%, 0%) | 0%  (0%, 0%) | 0%  (0%, 0%) |
| Fidelity | Did a team recently visit your community to distribute azithromycin? | 99.6%  (99.3%, 99.8%) | 99.3%  (98.8%, 99.7%) | 99.4%  (99.2%, 99.7%) |
|  | Do you know why azithromycin was distributed in your community? | 90.7%  (87.0%, 94.5%) | 92.1%  (88.6%, 95.4%) | 91.4%  (88.9%, 93.9%) |
|  | *For those who indicated knowing why the distribution was done:* why was azithromycin distributed in your community? |  |  |  |
|  | To reduce child mortality | 36.3%  (31.0%, 41.6%) | 37.4%  (30.5%, 44.2%) | 36.8%  (32.6%, 41.1%) |
|  | To reduce child illness | 63.5%  (58.2%, 68.8%) | 62.4%  (55.5%, 69.3%) | 62.9%  (58.7%, 67.2%) |
|  | Other | 0.2%  (0%, 0.5%) | 0.2%  (0%, 0.5%) | 0.2%  (0.06%, 0.4%) |
| Participation^2^ | | 96.8%  (94.7%, 99.0%) | 99.4%  (98.9%, 99.8%) | 98.1%  (97.0%, 99.2%) |

CI, confidence interval

^1^In the door-to-door arm, 58 caregivers indicated they did not participate and in the fixed-point arm, 71 indicated they did not participate.

^2^In the door-to-door arm, 47/3711 (1.3%) eligible caregivers did not participate in the survey and in the fixed-point arm, 6/3422 (0.18%) did not participate. Overall, 53/7133 (0.74%) eligible caregivers did not participate.

# Appendix Table 6. Full responses to stakeholder survey by domain for Community Health Workers. Responses shown as mean cluster-level summaries.

| **Domain** | **Survey Question** | **Door-to-Door**  **% (95% CI)** | **Fixed-point**  **% (95% CI)** | **Overall**  **% (95% CI)** |
| --- | --- | --- | --- | --- |
| Acceptability | Were the communities you worked in supportive of the program? | 88.9%  (76.2%, 100%) | 99.3%  (97.9%, 100%) | 93.9%  (87.2%, 100%) |
| Appropriateness | Do you think this is a useful intervention for your community? | 92.6%  (83.6%, 100%) | 100%  (100%, 100%) | 96.1%  (91.4%, 100%) |
|  | Which approach do think is better for communities, door-to-door delivery or fixed-point delivery? *Percentage of participants indicating preference for door-to-door shown.* | 97.4%  (93.4%, 100%) | 51.3%  (31.1%, 71.6%) | 75.3%  (63.7%, 86.8%) |
|  | *For those who responded with preference for door-to-door delivery:* Why do you prefer that approach? |  |  |  |
|  | The method makes administering azithromycin easier | 34.8%  (18.6%, 51.1%) | 32.7%  (14.1%, 51.2%) | 33.8%  (21.9%, 45.6%) |
|  | Less personal travel required to receive treatment | 9.3%  (0%, 18.8%) | 6.7%  (0%, 15.8%) | 8.0%  (1.6%, 14.4%) |
|  | The method is more able to ensure all children are treated | 44.7%  (26.2%, 63.2%) | 36.0%  (17.6%, 54.4%) | 40.5%  (27.8%, 53.2%) |
|  | The method avoids possible conflict | 3.8%  (0%, 8.5%) | 18.7%  (3.1%, 34.2%) | 11.0%  (3.1%, 18.8%) |
|  | The chef du village influenced my decision | 0%  (0%, 0%) | 1.3%  (0%, 4.1%) | 0.6%  (0%, 1.9%) |
|  | Other | 5.6%  (0%, 13.9%) | 4.7%  (0%, 13.0%) | 5.1%  (0%, 10.8%) |
|  | Which approach do you prefer as a community health worker, door-to-door delivery or fixed-point delivery? | 98.1%  (94.3%, 100%) | 51.3%  (31.1%, 71.6%) | 75.6%  (64.0%, 87.2%) |
|  | Are there other ways the program could be delivered that you would prefer? *Percentage of participants responding “Yes” is shown.* | 0%  (0%, 0%) | 0%  (0%, 0%) | 0% (0%, 0%) |
| Fidelity | Do you know why azithromycin was being distributed in your community? | 96.3%  (88.7%, 100%) | 100%  (100%, 100%) | 98.0%  (94.1%, 100%) |
|  | *For those who indicated knowing why the distribution was done:* why was azithromycin distributed in your community? |  |  |  |
|  | To reduce child mortality | 44.2%  (34.6%, 53.9%) | 43.2%  (30.4%, 56.0%) | 43.8%  (36.1%, 51.4%) |
|  | To reduce child illness | 55.8%  (46.1%, 65.4%) | 56.8%  (44.0%, 69.6%) | 56.2%  (48.6%, 63.9%) |
|  | Other | 0%  (0%, 0%) | 0%  (0%, 0%) | 0%  (0%, 0%) |
|  | Did the training provide you enough information to implement the protocol? | 96.3%  (88.7%, 100%) | 95.8%  (87.4%, 100%) | 96.1%  (90.6%, 100%) |
|  | Did the supervision provide enough support for your effort? | 100%  (100%, 100%) | 100%  (100%, 100%) | 100%  (100%, 100%) |
| Participation^1^ |  | 95.7%  (91.6%, 99.8%) | 94.2%  (87.3%, 100%) | 95.0%  (91.1%, 98.9%) |
|  |  |  |  |  |
| Fidelity – Supervisor^2^ | Was the intervention implemented according to protocol?^3^ | 84.4%  (66.2%, 100%) | 80.6%  (57.6%, 100%) | 82.7%  (69.3%, 96.1%) |
|  | Informed Consent | 91.1%  (78.1%, 100%) | 88.9%  (72.4%, 100%) | 90.1%  (80.6%, 99.7%) |
|  | Data Collection Form | 93.3%  (79.0%, 100%) | 100%  (100%, 100%) | 96.3%  (88.7%, 100%) |
|  | Dosage – Height | 100%  (100%, 100%) | 91.7%  (73.3%, 100%) | 96.3%  (88.7%, 100%) |
|  | Dosage – Weight | 100%  (100%, 100%) | 100%  (100%, 100%) | 100%  (100%, 100%) |
|  | Treatment Preparation | 91.1%  (78.1%, 100%) | 97.2%  (91.1%, 100%) | 93.8%  (86.5%, 100%) |
|  | Administration – Dosing Cup | 100%  (100%, 100%) | 100%  (100%, 100%) | 100%  (100%, 100%) |
|  | Administration Syringe | 100%  (100%, 100%) | 100%  (100%, 100%) | 100%  (100%, 100%) |

CI, confidence interval

^1^In the door-to-door arm, 3/47 (6.4%) eligible community health workers did not participate in the survey and in the fixed-point arm, 3/45 (6.7%) did not participate. Overall, 6/93(6.5%) eligible community health workers did not participate.

^2^Results shown for 48 completed supervisor visits, 26 in the door-to-door arm and 22 in the fixed-point arm.

^3^Numbers and percentages of community health workers correctly implementing all supervised protocol steps.

# Appendix Table 7. Full responses to stakeholder survey by domain for Community Leaders. Responses shown as mean cluster-level summaries.

| **Domain** | **Survey Question** | **Door-to-Door**  **% (95% CI)** | **Fixed-point**  **% (95% CI)** | **Overall**  **% (95% CI)** |
| --- | --- | --- | --- | --- |
| Acceptability^1^ | Was the program accessible? | 100%  (100%, 100%) | 100%  (100%, 100%) | 100%  (100%, 100%) |
|  | Was the amount of time involved in participation reasonable? | 100%  (100%, 100%) | 100%  (100%, 100%) | 100%  (100%, 100%) |
|  | Were the community health workers helpful? | 100%  (100%, 100%) | 100%  (100%, 100%) | 100%  (100%, 100%) |
| Appropriateness | Which approach do you prefer, door-to-door delivery or fixed-point delivery? Why? | 90.3%  (81.4%, 99.2%) | 72.4%  (57.7%, 87.0%) | 81.2%  (72.5%, 89.9%) |
|  | *For those who responded with preference for door-to-door delivery:* Why do you prefer that approach? |  |  |  |
|  | The method makes administering azithromycin easier | 17.1%  (5.1%, 29.2%) | 31.5%  (16.6%, 46.5%) | 23.5%  (14.1%, 33.0%) |
|  | Less personal travel required to receive treatment | 15.7%  (3.7%, 27.7%) | 10.1%  (1.0%, 19.2%) | 13.2%  (5.8%, 20.7%) |
|  | The method is more able to ensure all children are treated | 60.0%  (43.9%, 76.1%) | 49.4%  (33.2%, 65.6%) | 55.3%  (44.1%, 66.5%) |
|  | The method avoids possible conflict | 5.7%  (0%, 13.6%) | 8.9%  (0%, 17.9%) | 7.1%  (1.4%, 12.9%) |
|  | Other | 1.4%  (0%, 4.3%) | 0%  (0%, 0%) | 0.8%  (0%, 2.2%) |
| Fidelity | Was a special meeting held to ask for your consent for your community’s participation? | 99.1%  (87.3%, 100%) | 100%  (100%, 100%) | 99.6%  (98.7%, 100%) |
| Participation^2^ | | 100%  (100%, 100%) | 100%  (100%, 100%) | 100%  (100%, 100%) |

CI, confidence interval

^1^Acceptability questions were only asked of community leaders with eligible children who might have participated in the program. In the door-to-door arm, this included 27 leaders, in the fixed-point, 15 leaders, and overall, 42 leaders.

^2^In the door-to-door arm, 0/51 (0%) eligible community leaders did not participate in the survey and in the fixed-point arm, 3/48 (6.3%) did not participate. Overall, 3/99 (3.0%) eligible community leaders did not participate.

# Appendix Fig 1. Histograms of the distribution of Round 2 treatment coverage by arm.


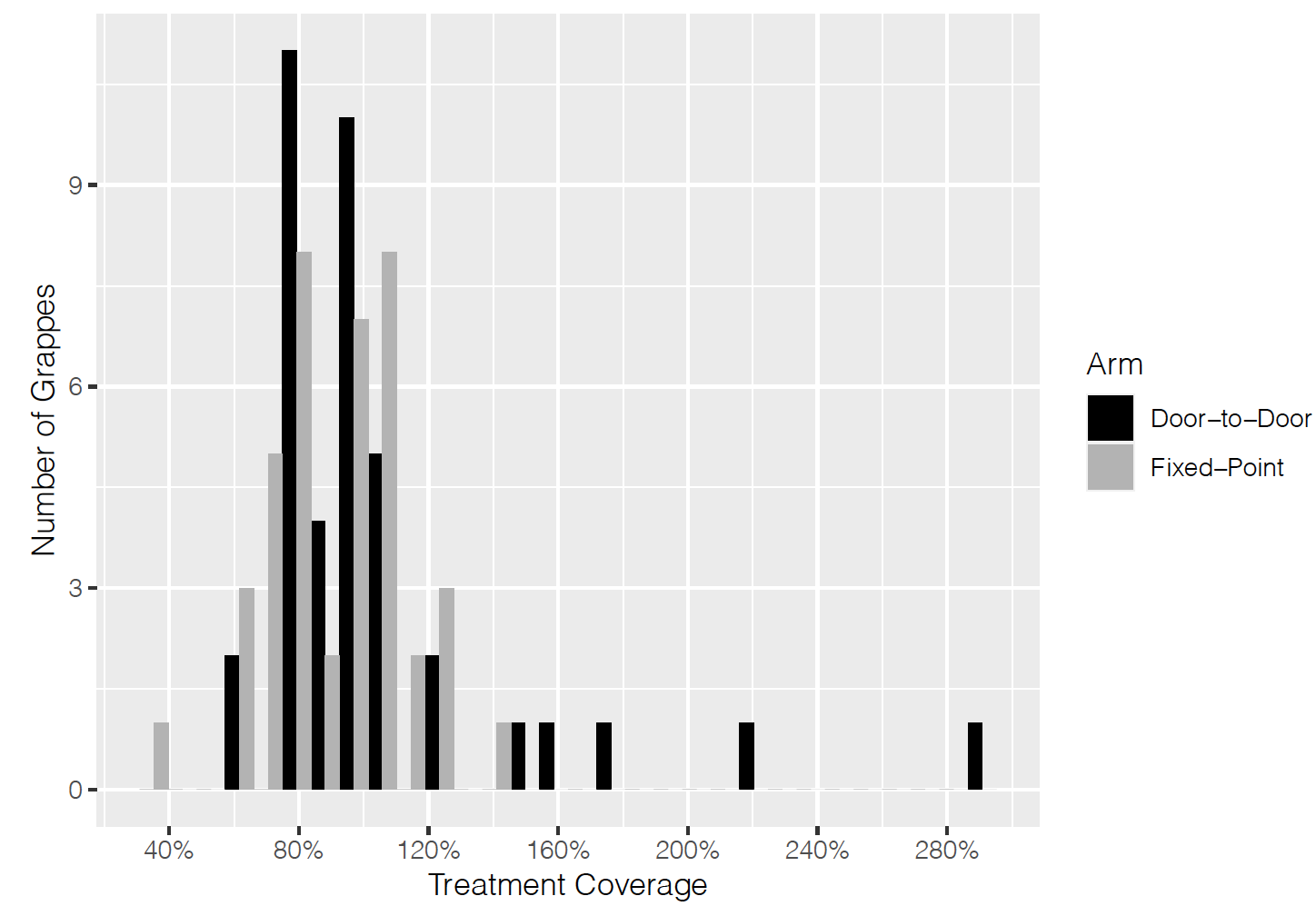


Appendix Fig 2. Boxplots of treatment coverage by community-level subgroups. Large population size defined as having 150 people or more, and far distance defined as being located 5 kilometers or more from the nearest health center.


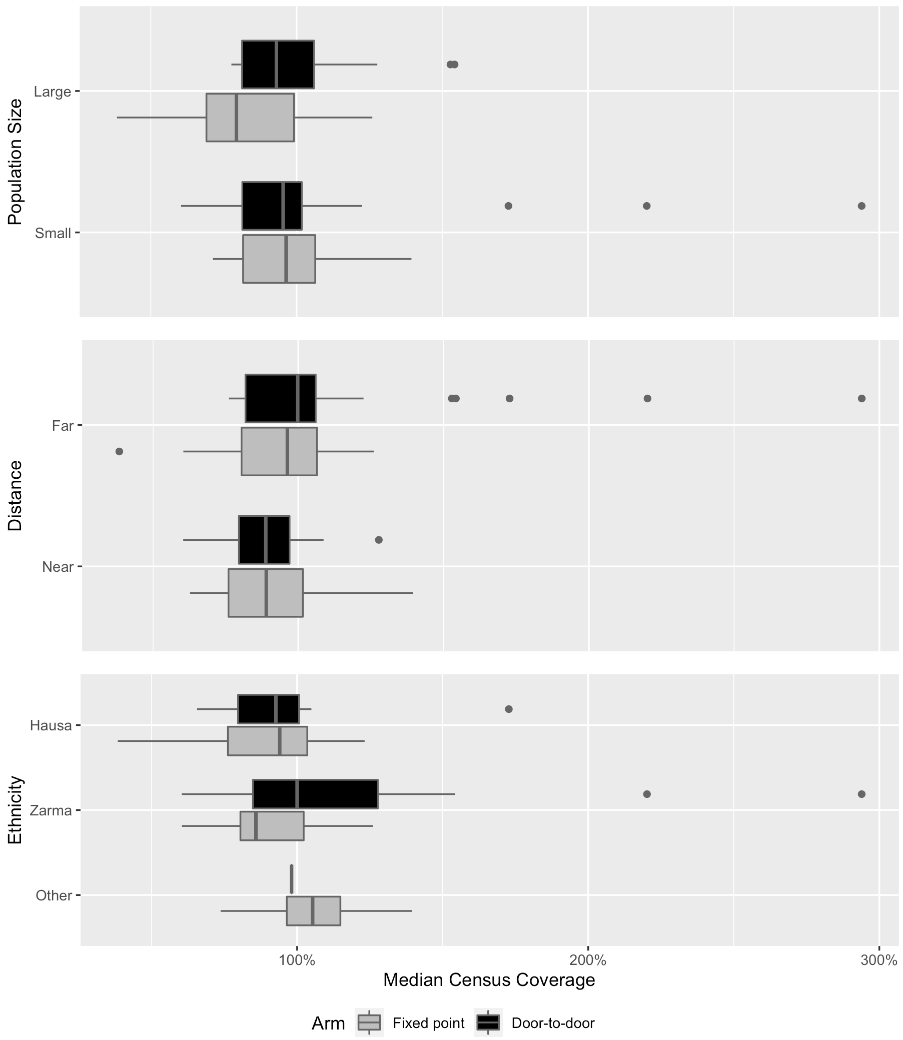


Appendix Fig 3. Boxplots of treatment coverage shown by individual level subgroups. Community health workers tracked the number of children treated by age group (1-11 months or 12-59 months) and sex (female or male) for each community.


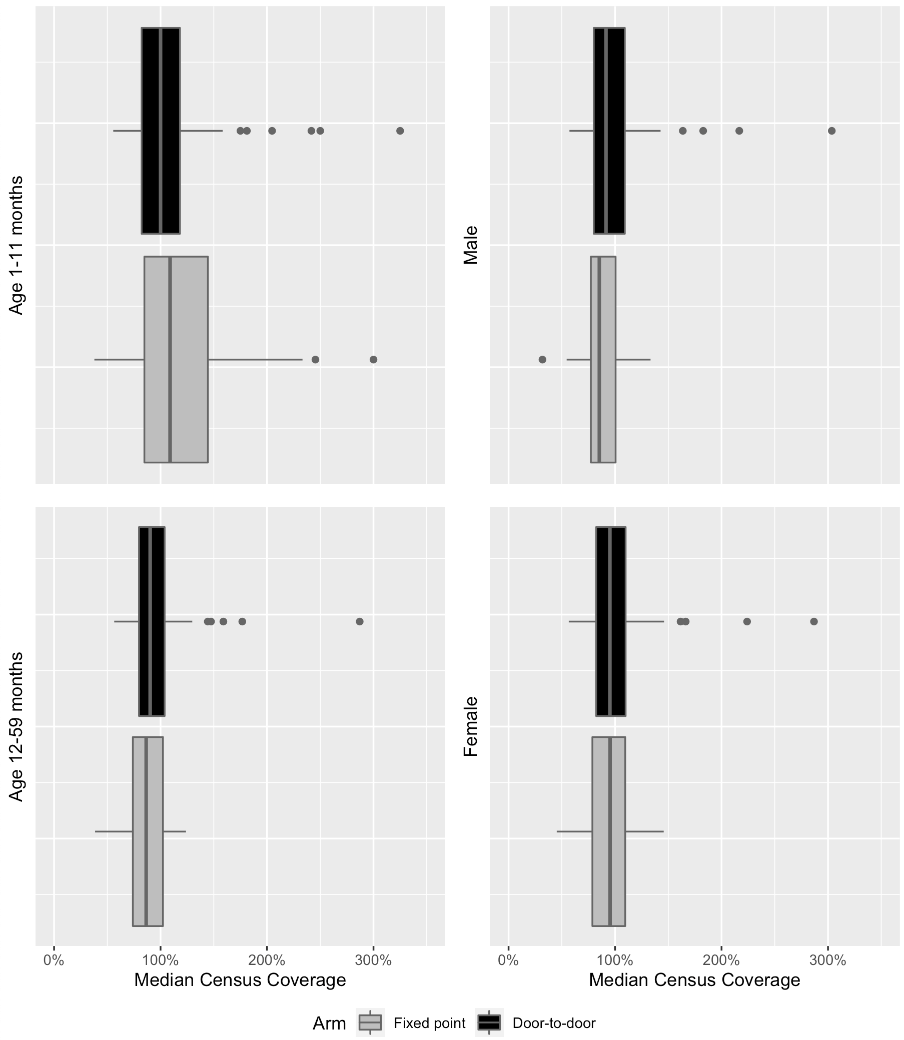

Supplement: S1 Appendix — (DOCX) [file pgph.0002559.s003.docx]
