# Supplementary material for: Comparison of door-to-door and fixed-point delivery of azithromycin distribution for child survival in Niger: A cluster-randomized trial
Source: PLOS Glob Public Health. 2023 Nov 15;3(11):e0002559. doi: 10.1371/journal.pgph.0002559 (PMC10651009; doi:10.1371/journal.pgph.0002559)
Supplement: S1 Questionnaire — (DOCX) [file pgph.0002559.s004.docx]

**S1 Questionnaire. PLOS Inclusivity in Global Research Questionnaire.**

**Ethical considerations, permits and authorship**

*This section is applicable to all research types.*

**Provide details as to who granted permissions and/or consent for the study to take place in the Methods section of your manuscript. This should include the names of all ethics boards, governmental organizations, community leaders or other bodies that provided approval for the study. If individuals provided approval refer to these people by their role or title but do not list their name(s).**

Reported on page number: 4-5 (Methods section, Ethics and oversight)

**If there were any deviations from the study protocol after approval was obtained please provide details of these changes in the Methods section of your manuscript.**

Reported on page number: NA

**Did this study involve local collaborators that are residents of the country where the research was conducted or members of the community studied? If you do not have any authors from said communities, please provide an explanation for this below.**

This study is a collaboration between the Niger Ministry of Health, the Centre de Recherche et Interventions et Sante Publique in Niger, and the University of California, San Francisco. The first 10 authors listed on the manuscript are Nigerien. The AVENIR Study Group document provides the full list of collaborators from all participating groups.

**Everyone listed as an author should meet PLOS’ criteria for authorship and all individuals who meet these criteria should be included in the author byline, rather than the acknowledgements. Authorship criteria is based on the International Committee of Medical Journal Editors (ICMJE) Uniform Requirements for Manuscripts Submitted to Biomedical Journals - for further information please see here: https://journals.plos.org/plosone/s/authorship.**

All authors meet the ICMJE criteria for authorship.

**Human subjects research (e.g. health research, medical research, cross-cultural psychology)**

**Did you obtain written informed consent from a representative of the local community or region before the research took place? How did you establish who speaks for the community? Details of written informed consent obtained from study participants should be reported separately in the Methods section of your manuscript.**

Informed consent for participation in this project took place at multiple levels in accordance with standard practices for this setting. National ethical approval was obtained from the Niger Ministry of Health. At the community level, verbal consent was obtained from community and local health center leaders before study activities commenced. At the individual level, informed consent was obtained from caregivers before study procedures were conducted, with written consent obtained for children 30-42 days old and verbal consent obtained for children older than 42 days. Written consent was required for the younger age group given the potential risk of macrolide-associated infantile hypertrophic pyloric stenosis that has been suggested in studies conducted in other settings

**How did members of the local community provide input on the aims of the research investigation, its methodology, and its anticipated outcome(s)?**

The aims and methods of this study were developed in collaboration between the Niger Ministry of Health, the Centre de Recherche et Interventions et Sante Publique in Niger, and the University of California, San Francisco through a series of in person meetings and design workshops along with regular online communication and meetings. Part of the aim of this project was to capture perceptions of community members (community health workers, community leaders, and caregivers of eligible children) about this intervention through surveys.

**When engaging with the local community, how did you ensure that the informed consent documents and other materials could be understood by local stakeholders?**

Sensitization and consent materials were prepared in French and were translated into local languages by trained members of the study team when communicating with local stakeholders, including Djerma, Hausa, and Peul.

**Will the findings of the research be made available in an understandable format to stakeholders in the community where the study was conducted (e.g. via a presentation, summary report, copies of publications, etc.)? Please provide details of how this will be achieved.**

Quarterly reports on progress and results within the larger AVENIR project are at multiple levels (national, regional, district) and include in written reports in French and presentations in local languages.

**Non-human subjects research using specimens/ animals collected as part of the study, or those housed in archival collections. Examples include archaeology, paleontology, botany and zoology.**

**Did the permission you obtained from a local authority to perform the study include an agreement on access to outputs and benefit sharing? This may include procedures to enable fair distribution of the benefits and resources arising from the research performed. Please include any details of Prior Informed Consent and Benefit Sharing Agreements obtained. These may be required by field-specific regulations, for example the Convention on Biological Diversity (CBD) and the associated Nagoya Protocol.**

NA

**If the material used in your study was imported, please A) provide the year it was imported and B) indicate whether permits were obtained to import/export the materials used, C) provide details of any permits obtained. If this information is not available, please indicate this.**

NA

**If you used archival specimens, please state how the material used in your study was acquired by the institute it is held in and provide details of any permits obtained for the original excavations/ sample collection. If this information is not available, please indicate this.**

NA

**How was the potential cultural significance of the materials collected in your study to local communities considered in your research design? Were Indigenous peoples and/or local researchers and institutions involved with archaeological excavations / collection of specimens? If so, please provide a description of their involvement.**

NA

**If your manuscript includes photographs of human remains please indicate whether authors obtained permission from descendants or affiliated cultural communities to do so.**

NA
