# Supplementary material for: Comparison of door-to-door and fixed-point delivery of azithromycin distribution for child survival in Niger: A cluster-randomized trial
Source: PLOS Glob Public Health. 2023 Nov 15;3(11):e0002559. doi: 10.1371/journal.pgph.0002559 (PMC10651009; doi:10.1371/journal.pgph.0002559)
Supplement: S1 File — (PDF) [file pgph.0002559.s005.pdf]

UCSF
University of California
San Francisco

Human Research Protection Program
Institutional Review Board (IRB)

Full Committee Approval

Principal Investigator
Dr. Thomas M Lietman

Co-Principal Investigator
Kieran O'Brien MPH

Type of Submission: Submission Response for Initial Review Submission Packet
Study Title: L' azithromycine pour la vie des enfants au Niger: implé mentation et recherche

IRB #: 19-28387
Reference #: 256124
Reviewing Committee: San Francisco General Hospital Panel
Study Risk Assignment: Greater than minimal

Approval Date: 01/30/2020 **Expiration Date:** 01/29/2021

Regulatory Determinations Pertaining to This Approval:
The IRB Committee agreed that an IND was not needed for the use of the drug/supplement in this study.

This research satisfies the following condition(s) for the involvement of children:

45 CFR 46.404, 21 CFR 50.51: Research not involving greater than minimal risk. This applies to all subjects over the age of 2 months.
45 CFR 46.405, 21 CFR 50.52: Research involving greater than minimal risk but presenting the prospect of direct benefit to the individual subjects. This applies to subjects 1-2 months of age.

Parental Permission and Assent:
The permission of one parent or guardian is sufficient.
The assent of the children will be obtained.

The research meets all of the conditions of 45 CFR 46.204 for the involvement of pregnant women or fetuses.

This research is not subject to HIPAA rules.

A waiver of the requirement to obtain a signed consent form is acceptable for subjects over the age of 2 months because, as detailed in the application, the research presents no more than

minimal risk of harm to subjects and involves no procedures for which written consent is normally required outside of the research context.
The waiver applies to subjects over the age of 2 months.
A waiver is not acceptable for subjects less than 2 months of age. Signed consent must be obtained.

IRB Comments: No work should begin on this study until all IRB approvals are in place.

All changes to a study must receive UCSF IRB approval before they are implemented. Follow the modification request instructions. The only exception to the requirement for prior UCSF IRB review and approval is when the changes are necessary to eliminate apparent immediate hazards to the subject (45 CFR 46.103.b.4, 21 CFR 56.108.a). In such cases, report the actions taken by following these instructions.
Expiration Notice: The iRIS system will generate an email notification eight weeks prior to the expiration of this study's approval. However, it is your responsibility to ensure that an application for continuing review approval has been submitted by the required time. In addition, you are required to submit a study closeout report at the completion of the project.

Documents Reviewed and Approved with this Submission:

Consent Documents

Study Consent Form			
Title	Version #	Version Date	Outcome
Written Consent for Participation in Census and 30-42d Treatment (Consent and Parental Permission) 10DEC19	Version 1.1	01/29/2020	Approved
Verbal Consent for Participation in Census and 1-59m Treatment (Consent and Parental Permission)	Version 1.3	12/10/2019	Approved
Verbal Consent for Participation in Swabs and Stool Collection (Community Leader Consent)	Version 1.2	12/17/2019	Approved
Verbal Consent for Swab and Stool Collection of Mothers (Adult Consent)	Version 1.2	12/17/2019	Approved
Verbal Consent for Swabs 1-59m 7-12y (Parental Permission)	Version 1.2	12/17/2019	Approved
Verbal Consent for Participation in Census and Treatment (Community Leader Consent)	Version 1.2	12/10/2019	Approved
Verbal Consent for	Version 1.2	12/17/2019	Approved

Swabs 1-59m CSI (Parental Permission)			
Verbal Assent for Swabs 7-12 (Child Assent)	Version 1.2	12/17/2019	Approved

Other Study Documents

Study Document			
Title	Version #	Version Date	Outcome
AVENIR MOP_v0.26	Version 1.0	07/02/2019	Approved

For a list of all currently approved documents, follow these steps: Go to My Studies and open the study – Click on Informed Consent to obtain a list of approved consent documents and Other Study Documents for a list of other approved documents.

San Francisco Veterans Affairs Medical Center (SFVAMC): If the SFVAMC is engaged in this research, you must secure approval of the VA Research & Development Committee in addition to UCSF IRB approval and follow all applicable VA and other federal requirements. The IRB website has more information.

REPUBLIQUE DU NIGER Niamey le 14 09 2020

MINISTERE DE LA SANTE PUBLIQUE
COMITE NATIONAL D'ETHIQUE
POUR LA RECHERCHE EN SANTE
CNERS

DELIBERATION N° 041/2020/CNERS

Objet : Autorisation

LE COMITE NATIONAL D'ETHIQUE POUR LA RECHERCHE EN SANTE **REUNI A NIAMEY le mardi 10 09 2020**

Vu la Constitution du 25 novembre 2010 ;

Vu la loi n° 2011-020 du 8 Août 2011 déterminant l'organisation générale de l'administration Civile de l'Etat et fixant ses missions ;

Vu le décret n°2013-504/PRN/MSP du 04 décembre 2013 portant organisation du Ministère de la Santé Publique ;

Vu le décret n° 2016-161/PRN du 02 avril 2016, portant nomination du Premier Ministre, Chef du Gouvernement ;

Vu le décret n° 2016-572/PRN du 19 0ctobre 2016, portant remaniement des membres du Gouvernement ;

VU le décret n°2016-623/PRN/MSP du 14 Novembre 2016 portant organisation du Gouvernement et fixant les attributions des Ministres d'Etat, des Ministres et des Ministres délégués ;

Vu le décret n°2016-624/PM du 14 Novembre 2016 précisant les attributions des membres du Gouvernement ;

VU le décret n°2016-644/PRN/MSP du 01 décembre 2016 portant création, missions, composition et fonctionnement du Comité National d'Ethique de la Recherche en Santé ;

Vu l'arrêté n°2017-000073/MSP/CAB/SG/DEP du 07 Février 2017 portant nomination des membres du Comité National d'Ethique pour la Recherche en Santé ;

Vu le protocole soumis en SEPT 2020 par PNSO « azithromycine pour la vie des enfants au Niger : implémentation et recherche (AVENIR)»

Après avoir entendu les membres de l'équipe d'investigation

DECIDE

Article Premier : PNSO est autorisé à conduire la recherche sur « azithromycine pour la vie des enfants au Niger : implémentation et recherche (AVENIR)» ;

Article 2 : Le promoteur doit finaliser le protocole final en tenant compte des observations formulées par le Comité lors de la session du **10 09 2020 ;**

Article 3 : Le promoteur doit soumettre au Comité les rapports d'étapes ainsi qu'une copie du rapport final ;

Article 4 : Le promoteur doit signaler au comité toutes difficultés majeures rencontrées sur le terrain ;

Article 5 : toute modification de ce protocole tel que soumis à la session du **10 09 2020 doit** faire l'objet d'une **nouvelle soumission** au comité d'éthique ;

Article 6 : pour les enquêtes internationales, impliquant les chercheurs nigériens leurs noms doivent figurer dans les publications des résultats ;

Article 7 : La présente décision sera communiquée partout où besoin sera.

Ampliations :

MSP ATCR
SG/MSP info
Chrono 1

Pour le Comité National d'Ethique

Vice Président CNERS

Mr JEAN ETIENNE IBRAHIM

Comité Ethique National Recherche Santé
CNERS NIGER

Republic of Niger
Ministry of Public Health
National ethical committee
CNERS

Niamey, September 14 2020

Deliberation N 041/2020/CNERS

Subject: authorization

The national ethical committee for health research met in Niamey on September 10 2020

According to the constitution of November 25, 2012

According to the law number 2011-020 from August 8, 2011, on the general organization of the civil administration from the government and its missions

According to the ordinance number 2013-504/PRN/MSP from December 4, 2013, on the organization of the ministry of public health

According to the ordinance number 2016-161/PRN from April 2, 2016, on nominating the prime minister chief of government

According to the ordinance number 2016-572/PRN from October 19, 2016, on reorganization of the government

According to the ordinance number 2016-623/PRN/MSP from November 14, 2016, on the organization of the government and its missions for the ministry and delegates

According to the ordinance number 2016-624/PM from November 14, 2016, on the government attributions

According to the ordinance number 2016-644/PRN/MSP from December 1, 2016, on the creation, missions, composition, and operation of the national ethical committee on health research

According to the bylaw number 2017-000073/MSP/CAB/SG/DEP from February 7, 2017, on the nomination of the members of the national ethical committee

According to the protocol submitted in September 2020 by the National program of eye health call “AVENIR: Azithromycine pour la vie des enfants au Niger”

After hearing the team of investigators

DECIDED

First article: The National program of eye health is authorized to conduct the study AVENIR

Second article: Final protocol will need to be submitted considering the comments made on September 10, 2020

Third article: Progress reports must be submitted as well as a final report

Fourth article: All difficulties encountered in the field must be communicated to the committee

Fifth article: modification to this protocol must be submitted to the ethical committee

Sixth article: For all international studies involving Nigerien researchers, their names must appear on published articles

Seventh article: this decision will be shared everywhere it is needed

Notarized:
MSP: ATCR
SG/MSP info
Chrono: 1

For the national ethical committee

Vice president of CNERS

Mr Jean Etienne IBRAHIM

Translated by Elodie Lebas, RN
UCSF FI Proctor Foundation
August 24, 2021
